# C-Reactive Protein and Cognition Are Unrelated to Leukoaraiosis

**DOI:** 10.1155/2014/121679

**Published:** 2014-01-22

**Authors:** Liara Rizzi, Fabricio Correia Marques, Idiane Rosset, Emilio Hideyuki Moriguchi, Paulo Dornelles Picon, Marcia Lorena Fagundes Chaves, Matheus Roriz-Cruz

**Affiliations:** ^1^Division of Geriatric Neurology, Service of Neurology, “Hospital de Clinicas de Porto Alegre” (HCPA), Ramiro Barcelos Street 2.350, 90035-903 Porto Alegre, RS, Brazil; ^2^Division of Gerontological Nursing, Faculty of Nursing, Federal University of Rio Grande do Sul (UFRGS), São Manoel Street 963, 90620-110 Porto Alegre, RS, Brazil; ^3^Department of Internal Medicine, Faculty of Medicine, Federal University of Rio Grande do Sul (UFRGS), Ramiro Barcelos Street 2.040, 90035-903 Porto Alegre, RS, Brazil

## Abstract

Elevated serum levels of C-reactive protein (CRP) have been associated with leukoaraiosis in elderly brain. However, several studies indicate that leukoaraiosis is associated with an increased risk of cognitive impairment. It is unknown how the effect of CRP on cognition is mediated by leukoaraiosis. The purpose of this study is to assess the relationship between serum levels of CRP, the presence of leukoaraiosis, and cognitive impairment in a population of coronary patients over 50 years old. CRP levels explained 7.18% (*P*: 0.002) of the variance of the MMSE. The adjustment for the presence of leukoaraiosis little changed this variance (5.98%, *P*: 0.005), indicating that only a small portion of the CRP influence on cognition was mediated via leukoaraiosis. Patients with CRP levels ≥5.0 had 2.9 (95% CI: 1.26–6.44) times more chance to present cognitive impairment (*P*: 0.012). We found that elevated serum levels of CRP were associated with increased risk of cognitive impairment in elderly and it was not mediated by presence of leukoaraiosis.

## 1. Introduction

Inflammation has been increasingly recognized as a component in cerebrovascular [1] and neurodegenerative diseases [2, 3]. In addition, biological aging of the brain is partly attributable to aging of the cerebrovascular circulation and the effects of vascular changes on the brain [4]. Inflammation has been linked to the pathogenesis of cardiovascular disease, obesity, and insulin resistance, which are so related to cognitive impairment [5]. The hypothesis that inflammation is related to cognitive impairment, although new, is consistent [2]. Therefore, few studies evaluated that circulating inflammatory proteins are associated with increased risk of dementia [6], cognitive impairment [7], and cerebral white matter lesions (WML), commonly referred to as leukoaraiosis [8–10].

CRP, composed of five 23 kDa subunits, is a hepatically derived pentraxin that has important role in the human immune system [11]. That protein is a sensitive nonspecific marker of systemic low-grade inflammation [5] and increased serum concentrations of CRP have been associated with impaired cognition, stroke, and depression [2, 6, 8]. Beyond proinflammatory response that causes neuronal damage directly, increased concentrations of CRP acting as cardiovascular risk factor—approved predictor by Food and Drug Administration—or causing brain atherosclerosis can result in cerebral macro- or microangiopathies. Both lesions can disrupt the integrity of frontal-subcortical circuits and are responsible for the development of cognitive impairment, dementia, or depressive disorders [12]. There are some pieces of evidence that elevated serum CRP levels may be a useful biomarker to identify individuals at an increased risk for cognitive impairment [7].

With the availability of improved brain imaging techniques, the high prevalence and clinical importance of cerebral small vessel disease have been increasingly recognized in recent years. WML are often found incidentally on image exams, predominantly in elderly people [13]. These WML reflect multiple physiologic and pathologic changes, including ischemic lesions, loss and deformation of myelin sheath, damage to the walls of small vessels, gliosis, micro-hemorrhages, and breaches of the cerebrospinal fluid brain barrier [14, 15]. All this damage may lead to an increase in the clinical consequences, namely, cognitive impairment, decreased mobility, and increased stroke risk [16]. Vascular risk, hypertension, and inflammation, which increase with age, may contribute to white matter deterioration and proliferation of WML; nonetheless, much white matter volume variance remains unexplained [10, 17]. Growing evidence shows that it is the cause of cognitive impairment and functional loss in the elderly population.

Whether inflammatory processes, excluded from their involvement in large-vessel disease, are implicated in the pathogenesis of cerebral small vessel disease remains unclear. Blood markers of vascular dysfunction reflect the underlying pathology and provide an independent measure of pathology based on biology. Some studies showed that systemic inflammatory processes, which include high levels of CRP, are related to the pathogenesis of cerebral small vessel disease at the development of cerebral WML and lacunar infarcts [9, 10, 17]. The increase of CRP at the microvasculature of the brain may act in synergy to promote arteriolosclerotic progression by different mechanisms like activation of classic complement system, mediation of low density lipoprotein uptake by macrophages, promotion of foam-cell formation, endothelial dysfunction, low nitric-oxide production, stimulation monotype recruitment, and vascular smooth muscle proliferation and migration [2]. All these processes cause narrowing of vascular lumen and failure of cerebral self-regulation, resulting in cerebral microangiopathies that may interrupt the integrity of the frontal-subcortical circuit and thus result in cognitive impairment [2, 9, 18]. The association of blood markers of vascular dysfunction with subclinical brain changes is still unclear.

Considering that high serum levels of CRP have been associated with leukoaraiosis and that several studies indicated that leukoaraiosis is associated with cognitive impairment, the aim of this study was to examine how much is the effect of CRP on cognition and if it was mediated by leukoaraiosis or not in a sample of coronary patients. We hypothesize that increased levels of the CRP biomarker would be related to brain leukoaraiosis, as evaluated by CT, and cognitive impairment.

## 2. Methods

The study population comprised outpatients assisted at the cardiology ambulatory of the Porto Alegre Clinics Hospital of the Federal University of Rio Grande do Sul, located in the southernmost state of Brazil. Patients with cognitive impairment were included in this study. All patients were 50 years or older. People who met the following criteria: dementia, stroke, Parkinson's disease, or other neurological diseases that potentially cause cognitive impairment, were excluded at the baseline.

The total sample was composed of 149 coronary patients, who were involved in the baseline examination and were assessed at the referred ambulatory. Subjects who obtained MMSE < 15 or CRP ≥ 13 were considered outliers to lower the probability that the associations could be attributed to subjects with apparent dementia (*n* = 14) totalizing 135 patients for the final sample. Patients with cognitive impairment were considered the cases (*n* = 34) and those without cognitive impairment were the controls (*n* = 101).

All analyses were adjusted for age, gender, and educational level. These variables were chosen for the analyses based on their known association with cognition as well as the association with CRP in the present study. Cognitive impairment increases exponentially with aging, and this reflects many biologic changes, including increase of inflammatory processes.

### 2.1. Sociodemographic Variables

A questionnaire was applied in order to assess the following sociodemographic data: income, age, sex, and years of schooling.

### 2.2. CRP Quantification

Patients were submitted to fasting venipuncture and the level of inflammatory protein CRP was assessed in serum samples, which were stored at −80°C until analyses. CRP concentration was determined with immunoturbidimetric assay (Siemens Healthcare Diagnostics Inc.) performed on a ADVIA 1800 Chemistry System (Siemens) that is based on the agglutination of latex particles when the CRP in the sample is coated with antihuman CRP antibodies. The degree of agglutination is proportional to the concentration of CRP in the sample and can be measured by turbidimetry (517 nm). This process is based on optical detection of very small particles suspended in liquid medium. When antihuman CRP antibody and sample are mixed they form immunocomplexes. Dilution acquires turbidity, which is proportional to the amount of antigen. The technicians were blinded to the clinical status of study participants and the samples.

### 2.3. Diagnosis of Leukoaraiosis by Computed Tomography Scans (CT Scan)

For each participant brain CT scan was performed using a CT imaging scanning (Philips Medical Systems). Trained neurologists and radiologists, who were blinded to patients, laboratorial and clinical data, assessed the existence, location, and extension of the leukoaraiosis lesions on CT scan. Images were analyzed by using a semiquantitative rating scale devised by Fazekas et al. [19]. This method scored subcortical and deep WML on a four-point scale of increasing severity as follows: 0: no lesions; 1: isolated hypodensities; 2: initially confluent hypodensities; 3: diffuse and extense hypodensities.

### 2.4. Assessment of Cognitive Function

To assess the cognitive function, the Mini Mental Status Examination (MMSE) [20] was performed to screen all subjects, translated, and validated for Brazilian population [21]. The MMSE is a tool that can be used to systematically and thoroughly assess mental status. It is a scale that tests seven areas of cognitive function: orientation to time/place, registration, attention/calculation, recall, language, and visual constructive capacity. The test was designed as a screening instrument for cognitive impairment and dementia and is widely used in both clinical practice and scientific studies. The score ranges from 0 to 30, with a higher score indicating better performance. A score of 23 or lower is indicative of cognitive impairment. Patients who scored less than minus two (≤−2) points from the expected MMSE score, corresponding to one standard deviation, were considered as cognitively impaired for the purpose of this study.

### 2.5. Assessment of Geriatric Depression

To assess depression symptoms, the Geriatric Depression Scale (GDS), developed by Yesavage et al. [22], was applied to the subjects. The GDS was translated and validated for Brazilian population [23]. The short form GDS was used consisting of 15 questions. Questions from the long form GDS which had the highest correlation with depressive symptoms in validation studies were selected for the short version. The short form is more easily used for patients who have short attention spans and/or feel easily fatigued. Presence of significant depressive symptomatology was considered for all subjects who scored ≥6 points in the scale.

### 2.6. Statistical Analysis

We examined the association between CRP and leukoaraiosis lesions by multivariate logistic regression analysis. The level of inflammatory protein was entered into the model through a linear term, in which the regression coefficient was expressed per standard deviation increase. A linear regression equation predicted the expected score of each person based on age, sex, and years of schooling (all *P* < 0.05). Student's *t*-test was used for independent variables. Analysis of covariance (ANCOVA) was used for adjusted means. Chi-square was used for categorical variables. All analyses were performed using Statistical Package for Social Sciences (SPSS), version 17.0.

### 2.7. Ethical Aspects

This study was approved by the Ethics Committee in Research of Porto Alegre Clinics Hospital (HCPA), project number 09-349. All participants provided informed consent. The experiments were undertaken with the understanding and written consent of each subject. This study was conducted in accord with the Declaration of Helsinki.

## 3. Results

The sociodemographic and clinical characteristics of the sample are summarized in Table 1 for numeric variables.  Table 2 shows characteristics for categorical variables. The mean age of the participants was 66.6 ± 8.7 years and the majority were men (60%). Foreseen MMSE was performed using the following equation and presented a variance of 35.6%:

(1) MMSE: 27.086−{[age×(−0.06)]  +[education  level×1.594]   +[gender×0.876]}.

Who obtained the difference between MMSE evaluated and foreseen ≤−2 (ΔMMSE ≤ −2) was considered with cognitive impairment, what means one standard deviation on this study. Thus, we analyzed 34 individuals with cognitive impairment (25.2%) and 101 individuals without cognitive impairment (controls).

**Figure 1:**
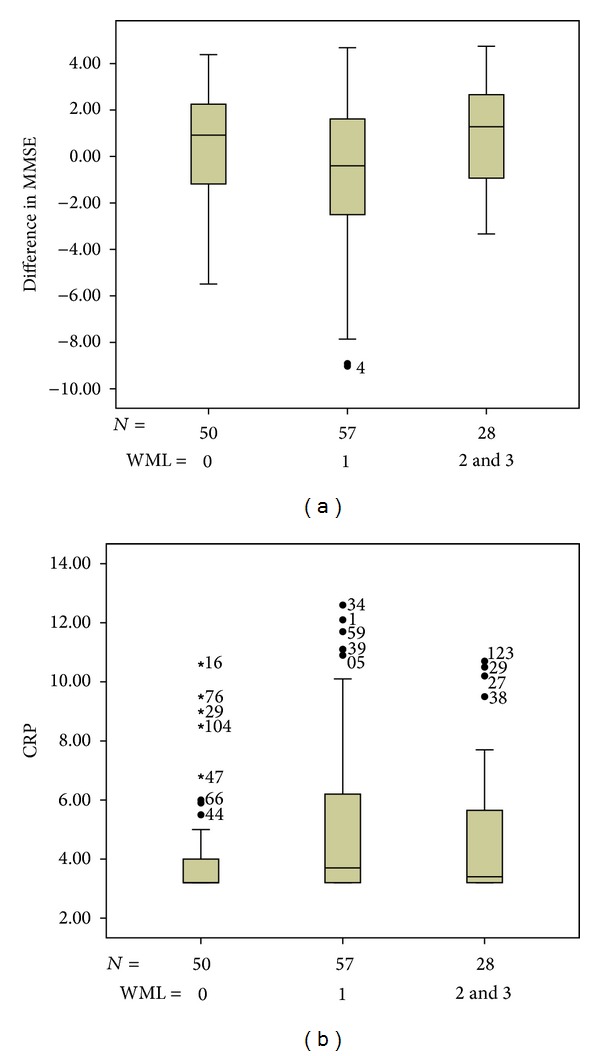
ANOVA between groups using a linear term; (a) *P*: 0.470 (b) *P*: 0.200. CRP: C-reactive protein; MMSE: Mini Mental Status Examination; WML: white matter lesions; 0: no lesions; 1: isolated hypodensities; 2: initially confluent hypodensities; 3: diffuse and extense hypodensities.

At first, we analyzed the degree of leukoaraiosis with ΔMMSE and CRP. We found no significant association between these items as showed on Figure 1. It suggests that the effects on the relationship between CRP and cognitive impairment are not completely mediated via leukoaraiosis.

85 patients (63%) were found to have any degree of leukoaraiosis on CT scan, while the remain 50 patients (37%) had no evidence of leukoaraiosis. Mean ΔMMSE was equal to −0.200 ± 3.24 for individuals with leukoaraiosis and +0.480 ± 2.7 for those without leukoaraiosis (*P* value: 0.250). Mean CRP levels equaled +5.070 ± 2.71 for subjects with leukoaraiosis and +4.080 ± 1.82 for those without leukoaraiosis (*P* value: 0.025).

**Figure 2:**
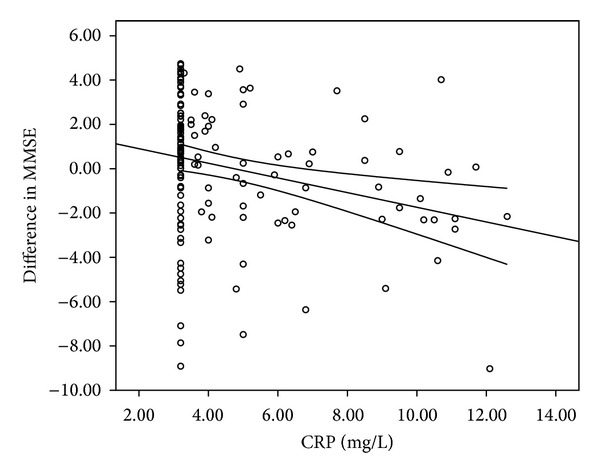
Linear regression showing the relationship between ΔMMSE and CRP levels (mg/L) (*P* < 0.001). CRP: C-reactive protein; MMSE: Mini Mental Status Examination.

Secondly, we analyzed the linear regression of the relationship between the ΔMMSE and CRP levels (mg/L), which means that high CRP levels are related to lower difference in the variation of MMSE (Figure 2).

**Figure 3:**
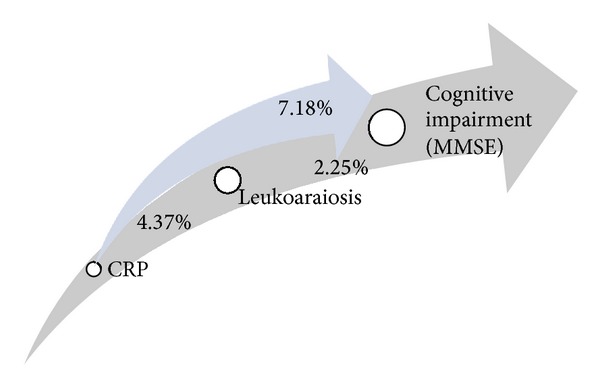
Relationship between the variance on cognition mediated by CRP levels (7.18%; *P*: 0.002) and adjusted for leukoaraiosis (5.98%; *P*: 0.005). CRP: C-reactive protein; MMSE: Mini Mental Status Examination.

With these results we found that the CRP levels can explain 7.18% (*P*: 0.002) of the variance of ΔMMSE, and adjusting for leukoaraiosis this variance little changed (5.98%; *P*: 0.005), showing that little CRP influence on cognition was mediated by leukoaraiosis, as evaluated by CT scan. Adjusted logistic regression analysis revealed that people with high levels of CRP had 2.9 (95% CI: 1.26–6.44) higher chance to present cognitive impairment (Figure 3).

**Figure 4:**
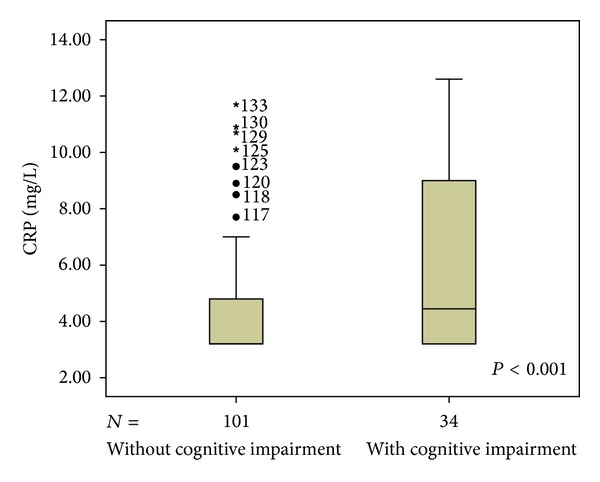
CRP level distribution in those without and with cognitive impairment. Mean CRP with cognitive impairment: 5.81; mean CRP adjusted for covariates: 5.7; *P* < 0.001. Mean CRP without cognitive impairment: 4.33; mean CRP adjusted for covariates: 4.7; *P* < 0.001. CRP: C-reactive protein.

**Figure 5:**
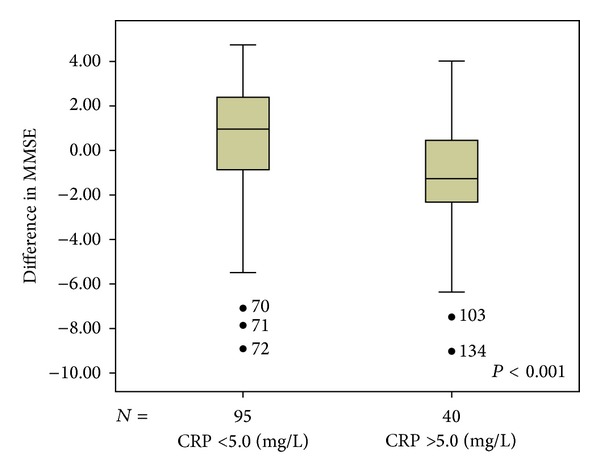
ΔMMSE distribution in those with high (≥5.0 mg/L) and normal (<5.0 mg/L) CRP levels. Mean ΔMMSE (CRP ≥ 5.0 mg/L) = −1.19, *P*: 0.003, and that adjusted for covariates: −1.13, *P*: 0.031 (not shown). Mean ΔMMSE (CRP < 5.0 mg/L) = +0.52, *P*: 0.003, and that adjusted for covariates: +0.30, *P*: 0.031 (not shown). CRP: C-reactive protein; MMSE: Mini Mental Status Examination.

We found 34 individuals with cognitive impairment, corresponding to 25.3% of sample. People that obtained CRP serum levels ≥5.0 mg/L were considered to be with a high CRP levels; we found 40 individuals with high CRP levels, corresponding to 29.8% of sample (Figures 4 and 5).

CRP levels between patients with cognitive impairment were significantly higher (5.82 ± 3.21) than among controls (4.33 ± 2.02; *P*: 0.002). Assessing Pearson's partial correlation coefficients, controlling for age, sex, and educational level, we found negative correlation between CRP and MMSE (*r*: −0.268; *P*: 0.002) and positive correlation between CRP and presence of leukoaraiosis lesions on CT (*r*: 0.209; *P*: 0.017). These results agree with the hypothesis that high levels of CRP are associated with cognitive impairment and that patients with WML have elevated serum levels of CRP.

## 4. Discussion

The present study found an inverse linear association between CRP marker and cognitive performance in a sample of patients with ischemic heart disease. We found that elevated serum levels of CRP were associated with worse cognitive function and an increased risk of cognitive impairment in people aged 50 and older. CRP levels in patients with cognitive impairment were significantly higher (5.82 ± 3.21) than among controls (4.33 ± 2.02; *P*: 0.002). Analyzing the degree of leukoaraiosis with ΔMMSE and with CRP we found no significant difference, which suggests that the effects on the relationship between CRP and cognitive impairment are not completely mediated via leukoaraiosis. These results are in accord with the hypothesis that high levels of CRP were associated with cognitive impairment and that patients with white matter lesions have elevated serum CRP levels. Moreover, the variance of CRP serum levels upon cognition (7.18%; *P*: 0.002) was independent of the degree of leukoaraiosis, because this variance little changed after adjustment for the later (5.98%; *P*: 0.005). These results remained significant even after accounting for confounders like age, sex, and educational level.

Thereby, our findings are in line with other studies that found that elevated CRP levels are related to cognitive impairment [24, 25]. A study found that elevated CRP levels predate by 25 years the clinical onset of dementia, suggesting that inflammatory process occurs long before clinical symptoms appear [8]. However, in some other studies no association between CRP and cognition was found [26–28].

The mechanisms underlying WML are not fully understood, but the observation that CRP, as a marker of inflammation, may be involved in the pathophysiology of cerebral small vessel disease is in accord with studies that link hypertension and diabetes to vascular dementia and small vessel subtypes [6]. Several studies have reported that some inflammatory proteins and cytokines are related to an increased risk of WML through endothelial dysfunction [9, 10, 29–31], whereas others have found no difference in the degree of WML according to inflammatory status [30, 32–34]. Diversity in the methods utilized may explain some of these variations among different studies.

Cerebral small vessel disease is one of the most common degenerative vessel disorders in the ageing of human brain, together with cerebral atherosclerosis and cerebral amyloid angiopathy [35]. Endothelial dysfunction is thought to play an important role on the cerebral small vessel disease and may be related to the pathogenesis of Alzheimer's disease and vascular dementia [36]. Therefore, elevated serum levels of CRP as endothelial biomarker dysfunction might contribute to the development of those pathologies or be a consequence of their injury. Finally, the disruption of subcortical neural circuits that control executive cognitive functioning leads to damage on short-term memory, organization, mood, regulation of attention, ability to act or make decisions, and appropriate behavior.

The population-based Rotterdam Scan Study evaluated 1033 nondemented elderly individuals and showed that higher CRP levels were associated with presence and progression of leukoaraiosis, independent of cardiovascular risk factors and the degree of carotid atherosclerosis [9]. This finding was later confirmed not only in whites but also among blacks as well in the Cardiovascular Health Study [29]. However, several studies failed to find associations between CRP and WML, especially in Asian populations [32, 33]. Asian populations seem to have low levels of CRP, which may reflect their low prevalence of coronary heart disease in comparison with those of Western populations [33]. Our analysis demonstrated that only a small portion of the CRP influence on cognition was mediated via leukoaraiosis. Studies showed that if the level of CRP is associated with cerebral small vessel disease, this protein might be utilized as a useful marker for monitoring the risk of cerebral small vessel disease-related brain lesions [33].

A meta-analysis that evaluated associations between CRP and cognitive deficit found that older men are more susceptible to elevated concentrations of CRP than elderly women [37]. Another study found that men are generally more susceptible to the deleterious effects of inflammation than women as suggested by the finding that CRP was associated with a 12% reduction in survival time and a one-year reduction in expected lifespan in men but not in women [38]. Therefore, gender may be an important variable to consider when studying the association between inflammation and cognition.

The levels of CRP in the brain are generally more than 100 times lower than in plasma [6, 39]. Increased plasma levels of proinflammatory proteins before the onset of clinical signs of dementia suggest that peripheral inflammation is involved in the disease process, which culminates in dementia. On the other hand, high concentrations of proinflammatory proteins in plasma may be a consequence of the dementia pathophysiology, because amyloid plaques induce the expression of cytokines, like IL-1 and IL-6, which increases the levels of peripheral proinflammatory proteins. This was demonstrated in animal models [40]. Thus, peripheral immune system activation might be both a cause and a consequence of the dementing process. This cascade includes the formation of beta-amyloid deposits and leads to local inflammation on the brain. This results in peripheral immune system activation, which, in turn, fosters increased deposition of beta-amyloid [6].

In this study, CRP levels are higher among patients with cognitive deficits and individuals with CRP levels ≥5.0 had 2.9 (95% CI: 1.26–6.44) times more chance to present cognitive impairment (*P*: 0.012). However, because this was a cross-sectional study, it is not possible to determine if elevated CRP levels occur before the development of dementia or are a consequence of the disease. That is, the cross-sectional design limits causal inferences and does not enable cause-effect inferences.

Other limitations of the study need to be taken into account. Measurement of the CRP was performed at only one time. Even though it is well known that CRP levels have few short-term fluctuations [41], within-person variability and measurement error may have resulted in dilution of the associations. Another limitation of our, as well as of most other studies, is that the inflammatory parameters measured in the circulation do not necessarily reflect local inflammation in the brain. Although CT scan is less sensitive than magnetic resonance image (MRI) for both the detection and quantification of leukoaraiosis, it is a more readily accessible method in developing countries than MRI.

Strength of our study includes simultaneous measurement of inflammatory biomarker CRP and white matter damage as assessed by CT scan in a population of high risk for small vessel cerebrovascular disease. The degree of leukoaraiosis was conducted by a blind rater using a previously validated method. Despite using low sensitivity methods like CT scan and MMSE, our findings were statistically significant. This probably means that if we have utilized more sensitive techniques the found associations would be even stronger.

Longitudinal studies would expect to find steeper rates of neural and cognitive impairment in people carrying higher levels of proinflammatory proteins, like CRP. Certain risk factors for cognitive impairment appear modifiable, and CRP represents a potentially modifiable inflammation marker that may be associated with an increased risk of cognitive impairment. Further research on the relationships among biomarkers, cognition, and structural brain changes in older adults is necessary in order to clarify the longitudinal associations between these variables.

One approach could be the use of functional image methods, like positron emission tomography scan (PET-scan), in order to evaluate the relationship between high CRP levels, often associated endothelial dysfunction, and chronic brain hypoperfusion that may lead to cognitive impairment before WML because small vessel disease can be noted on MRI. In particular, future studies need to investigate the mechanisms by which CRP is related to WML and worse cognition.

## 5. Conclusion

We found that CRP levels are inversely associated with cognitive performance in coronary patients and this relation was independent of age, sex, educational attainment, and degree of leukoaraiosis. Patients with CRP levels ≥5.0 had 2.9 (95% CI: 1.26–6.44) times more chance to present cognitive impairment (*P*: 0.012) than controls.

## Figures and Tables

**Table 1:** Student's *t*-test for numeric variables.

	Cognitive impairment (*n* = 34) Mean ± SD	Controls (*n* = 101) Mean ± SD	*P* value
Age	66.59 ± 8.1	66.56 ± 8.9	0.050
Years of schooling	2.06 ± 1.3	2.39 ± 1.2	0.047
GDS	4.68 ± 3.4	3.87 ± 3.1	0.034
Income	4.97 ± 1.3	4.95 ± 1.3	0.091
CRP (mg/L)	5.81 ± 3.2	4.33 ± 2.02	0.001
Leukoaraiosis score	0.88 ± 0.69	0.89 ± 0.88	0.003

GDS: Geriatric Depression Scale; CRP: C-reactive protein.

**Table 2:** Chi-square for categorical variables.

	Cognitive impairment (*n* = 34) *n* (%)	Controls (*n* = 101) *n* (%)	*P* value
Sex			
Male	19 (55.8%)	62 (61.4%)	0.057
Female	15 (44.2%)	39 (38.6%)	
GDS			
≤5	21 (61.7%)	68 (67.3%)	0.055
≥6	13 (38.3%)	33 (32.7%)	
CRP			
<5.0 mg/L	18 (52.9%)	77 (76.2%)	0.001
≥5.0 mg/L	16 (47.1%)	24 (23.8%)	
Leukoaraiosis			
No	9 (26.4%)	40 (39.6%)	0.002
Yes	25 (73.6%)	61 (60.4%)	

GDS: Geriatric Depression Scale; CRP: C-reactive protein.
